# Supplementary material for: Chromothripsis during telomere crisis is independent of NHEJ, and consistent with a replicative origin
Source: Genome Res. 2019 May;29(5):737–49. doi: 10.1101/gr.240705.118 (PMC6499312; doi:10.1101/gr.240705.118)
Supplement: Supplemental Material [file supp_gr.240705.118_Supplemental_file_1.zip › contigs/annotated_contigs/DB51/contig.4.DB51_length_1041_mean_cov_7.10854947166.docx]

**DB51_length_1041_mean_cov_7.10854947166**

```
TTGGTGAGGATGTGGAGCAAAGAGAATCCTTTTACACTATAGGTGGAAATTTAAATTGGTACAACCACTATGGAAATTAATATGGAGGT
 >chr6:79278280-79278612 + E=1e-184 p=5e-03
CCAAATAATCAAAAATGGAATTACTCTTTGATCCAGCAATTTCACTACTGCGTATATCCAAAAGAATTAAAATCTGGATTTCAAAAAGA

TATCTGTACTCCCATGTTTATTGCAGCATTATTCACAATAGTCAAGATATGAAAACAATCTAAGTATCTGTTGATGAATAAATGAATAT

GGAAAATGTGGTGTCGTTTATATACAATAGAATACTATTCAATGTTAAGAAAAAAGT|AATTTCTG|TGGCATGGAAGTTTAAATCTTA
                                                          >chr6:78571219-78571261 - E=2
TTAAATGCCTGA|CAAAGTAATTTATAGATTCACTGCCATCCCCATCAAGCTACCAGTGACTTTCTTCACAGAATTGAAAAAAAACTAC
e-13          >chr6:79280922-79281122 + E=2e-103 p=3e-03
TTTAAAGTTGATATGGAACCAAAAAAGAGCCCACATTGCCAAGACAATCCTAAGCAAAAAGAAAAAAGCTGAGGGCATCATGCCACCTG

ACTTCAAACTATACAACAAGGCTACGTAACCAAA|CTAATAATAAATAATATTTAAT|AAAATCTGCTTCAGGCACAGAGTACTTGGAT
                                                         >chr6:78571403-78571794 + E=4e
GAACTTAAGCAAGTTAAATTACTCTAAGACTCAGTTTTATCATTTGTGAAATTATTGTGAGGATTAATGATGTAGTACATATGAAGTGC
-220
TTAGTCCAGTATCTGACACACAGTAAATACTTCATGATAGTTCTTGTATTGTTGTTATTCTTACTGGTAGACATAATATTTCTGATGAT

CTTTTTTTATAATTAGACCATATGGTTGTTTGTTATTATTATCATGCATACTTCAAACCTGTCTAGATGAAACATCTTTTAACCAAACA

CATTTTGGGAAACATCATTCTAGGACATGTTTTGTAGCCAGTGAGTATCAAAAGCCTTGGTAGATCCACAGCAAAGAATTAAAAAAAAA

AAAA|GGGTGGGGGGGGGCAAGCAGGCCGAATTGACACGAGCGCAGTGGAAAGCGGTCGAGGGGTGGG
```
